# Anaphylaxis in middle-aged patients 

**DOI:** 10.5414/ALX02216E

**Published:** 2021-03-23

**Authors:** Wojciech Francuzik, Magdalena Kraft, Kathrin Scherer Hofmeier, Franziska Ruëff, Claudia Pföhler, Regina Treudler, Roland Lang, Thomas Hawranek, Nicola Wagner, Margitta Worm

**Affiliations:** 1 Allergy and Immunology, Department of Dermatology, Venereology and Allergology, Charité – Universitätsmedizin Berlin, Berlin,; 2 Central Emergency Department, University Hospital Halle (Saale), Martin Luther University Halle-Wittenberg, Halle (Saale), Germany; 3 Allergology, Clinic for Dermatology, University Hospital Basel, Basel, Switzerland,; 4 Clinic and Polyclinic for Dermatology and Allergology, University Hospital Munich, Munich,; 5 Clinic for Dermatology, Venerology and Allergology, Saarland University Hospital and Medical Faculty of Saarland University, Homburg,; 6 Clinic and Polyclinic for Dermatology, Venerology and Allergology, Leipzig University Hospital, Leipzig, Germany,; 7 University Clinic for Dermatology and Allergology, Paracelsus Medical Private University Salzburg, Salzburg, Austria and; 8 Dermatology Clinic, Erlangen University Hospital, Friedrich-Alexander University Erlangen-Nuremberg, Erlangen, Germany

**Keywords:** anaphylaxis, anaphylactic reaction, severe allergic reaction, middle age, venom allergy, food allergy, drug allergy

## Abstract

Age is one of the most important factors influencing the course of anaphylaxis: moreover, the frequency of elicitors of anaphylaxis is age-associated. We analyzed 8,465 anaphylactic episodes in adult patients in three age groups with a focus on patients in the middle-age group (35 – 65 years old). Insect venom was the most frequent trigger in this age group (51.2%) followed by drugs (22.8%) and food (17.3%). Severe reactions were observed in 40.1% of middle-aged patients and occurred more frequently in this age group than in patients below 35 years (27.6%) and less frequently than in patients over 65 years (55.6%). The symptoms and comorbidity profile also changed with age, most significantly regarding the increase in rates of concomitant cardiologic diseases and (severe) cardiovascular symptoms.

## Introduction 

Anaphylaxis is defined as a generalized, systemic hypersensitivity reaction of the immediate type that can occur at any age. Although each reaction has its individual course, age has a significant influence on the occurring symptoms and severity of anaphylaxis. Moreover, the spectrum of triggers differs significantly with age. Children react primarily with severe respiratory symptoms due to their relatively narrow airways [1]. In the pediatric population, the proportion of atopic patients is high so that allergies to ubiquitous foods are common. During the course of life, the number of somatic mutations increases (for example, the KIT V816D mutation) so that, in addition to IgE sensitization, mast cell-intrinsic mechanisms gain importance in the pathogenesis of anaphylaxis [2]. Finally, certain diseases present more frequently in higher age groups, e.g., arteriosclerotic changes in vessels and a previously damaged heart, which may aggravate severe cardiovascular symptoms [3] in case of a sudden mast cell degranulation with consecutive fluid shift into the third space due to a less efficiently compensation. 

Middle age is not a biologically defined term. In fact, the definition of this phase of life depends on social context. Due to a long period of education, youthful leisure, and consumeristic behavior of young adults, postponed family formation, later retirement and, last but not least, longer life expectancy, this phase of life has shifted significantly over the last decades [4]. International comparisons also show that middle age is defined very differently: in Saudi Arabia old age starts at 55 years, in Germany at 62 years, and in Spain at 74 years [5]. In this work, we defined the period of life between the ages of 35 and 65 as middle age and studied the triggers, course, and symptoms of anaphylaxis at this stage of life in comparison with young adulthood and older age. 

## Methods 

### Data set 

The Anaphylaxis Registry retrospectively collects information on moderate and severe anaphylactic reactions [6]. The registry was established in 2007 and originally included German-speaking centers. Since 2011, centers from non-German speaking countries have also contributed to the registry. Currently, more than 100 specialized tertiary allergy centers from 10 European countries and Brazil submit data on anaphylaxis cases to the registry. Pseudonymized data regarding the trigger, course, cofactors, and treatment of anaphylaxis was collected in a standardized manner via an internet-based questionnaire. Patients’ standard demographic information and pre-existing conditions were also recorded. Entries were based on the patient’s medical record (including records from the emergency department, if available) after completion of the diagnostic workup. The study has received a positive opinion from the Charité Ethics Committee and from the responsible ethics committees of all participating centers. 

### Patients 

**Figure 1:**
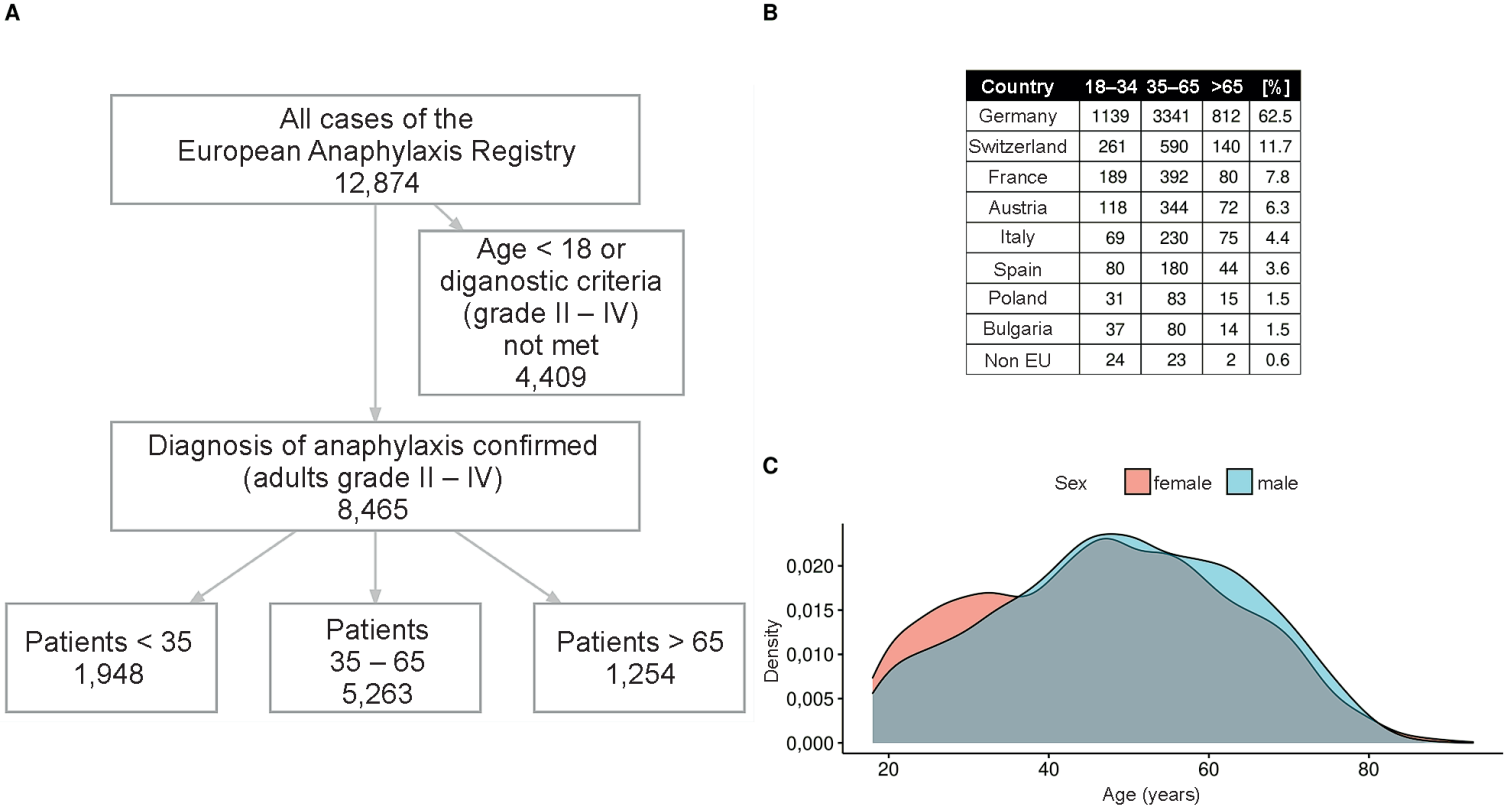
A: Flowchart of dataset cleaning. B: Age distribution in different countries. For each age category, the number of cases from each country is given. The last column shows the percentage of cases from the respective country (independent of age). C: Age distribution of women (red) and men (blue).

Between July 2007 and March 2019, 12,874 anaphylaxis cases were recorded in the registry. 3,643 reactions had occurred in children and 9,231 reactions in adults. Of the reactions in adults, 8,465 met the criterion of moderate to severe anaphylaxis (defined as severity according to Ring and Messmer ≥ 2 [7]) and were considered for further analysis (Figure 1A). 

### Statistical analysis 

The R statistical package was used for statistical analysis [8]. The normal distribution of the variables was checked using Shapiro-Wilk’s tests. Metric variables were mapped as mean ± standard deviation. The distribution of categorical variables was presented in absolute numbers and percentages. Differences in the distribution of categorical variables were tested for statistical significance using χ^2^-tests with Bonferroni-Holm correction. p-values < 0.05 were considered statistically significant. 

## Results 

### Mean age predominates in reported anaphylactic reactions 

Among the 8,465 patients with moderate and severe reactions in adults, 1,948 (23%) were younger than 35 years, 5,263 were middle-aged (62.2%; 35 – 65 years), and 1,245 were older than 65 years (14.7%). The median age was 50 years (43 – 57 years) (Figure 1A). Most patients were from Germany (62.5%), followed by Switzerland (11.7%), France (7.8%; including fewer cases from Belgium and Luxembourg), Austria (6.3%), Italy (4.4%), Spain (3.6%), Poland (1.5%), Bulgaria (1.5%), and Brazil (0.6%) (Figure 1B). With regard to age distribution, there were no significant differences between both sexes (t-test p-value 18 – 34 years old = 0.5412; 35 – 65 years old = 0.2890; > 65 years old = 0.5412) (Figure 1C). 

**Figure 2:**
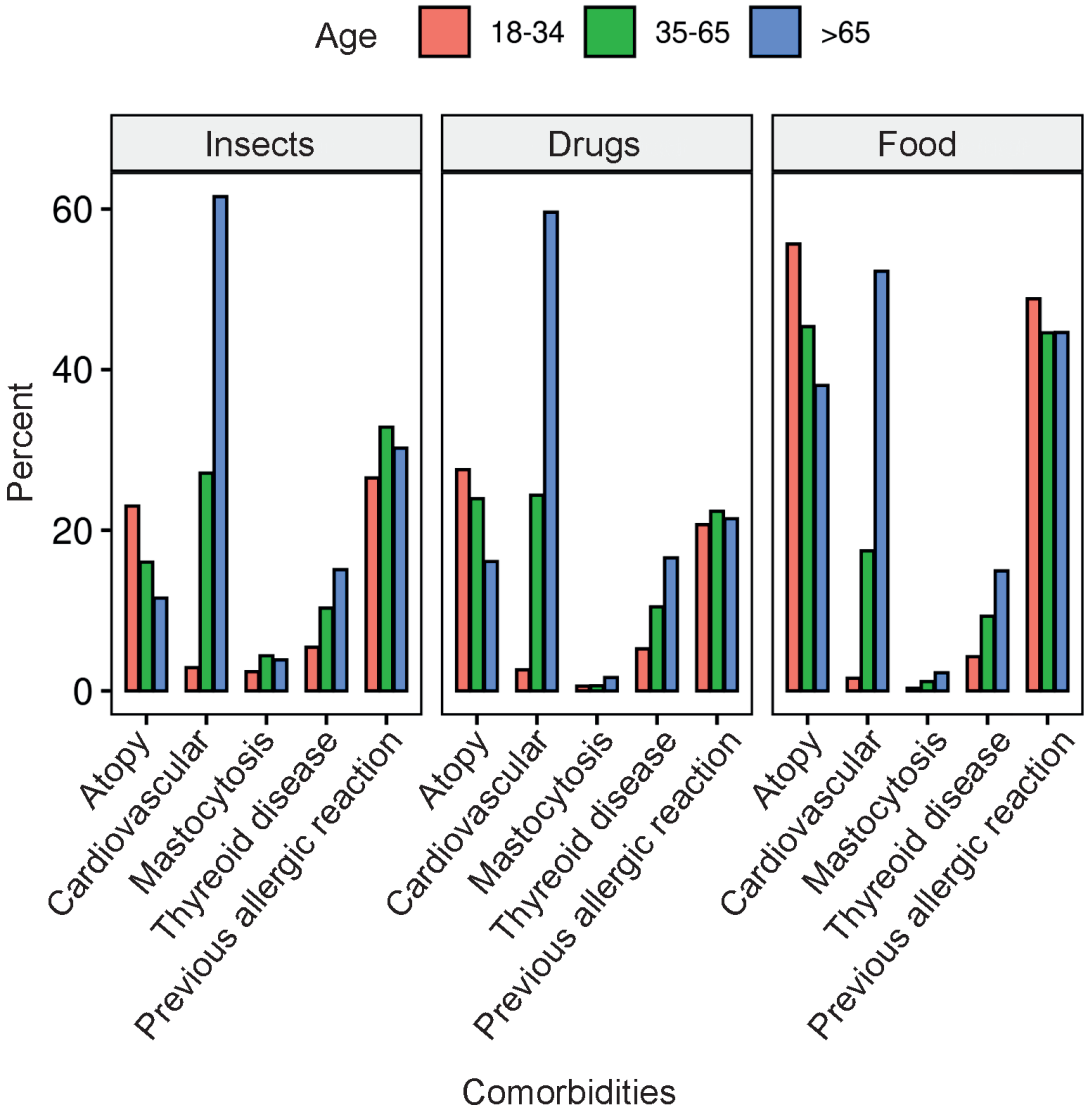
Frequency (in percent) of atopic (defined as allergic rhinoconjunctivitis, allergic asthma, or atopic dermatitis), cardiovascular, and thyroid diseases, or mastocytosis and previous allergic reactions (of any type) to the same trigger among patients of different age groups. Shown separately for insects (left), drugs (center), and food (right) as triggers of anaphylaxis.

Pre-existing atopic conditions (defined as allergic rhinoconjunctivitis, allergic bronchial asthma, and atopic dermatitis) were found mainly in patients with anaphylaxis to food (45.4%) and less in patients with reactions triggered by insects (16%) or drugs (23.92%) (Figure 2). History of cardiovascular disease, on the other hand, showed no correlation with the trigger but a strong age dependence: 2.2% of those younger than 35 years, 24.5% of those 35 – 65 years, and 59.9% of those older than 65 years had a history of cardiovascular disease. The prevalence of thyroid disease also increased with age and was not associated with a specific trigger. 

**Figure 3:**
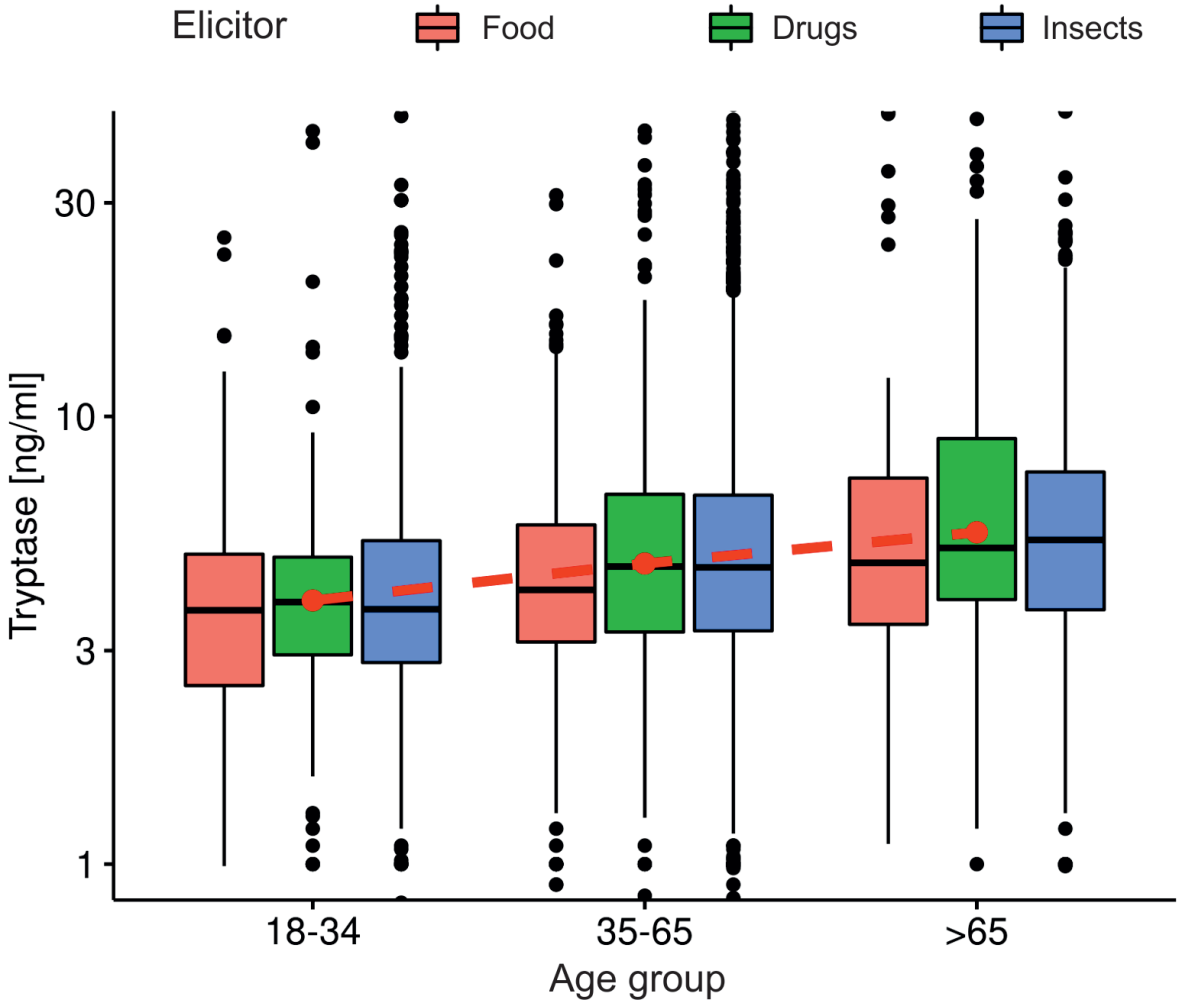
Basal tryptase level according to age group and trigger. The red dots show average values for each age group regardless of the trigger. The Y axis is a logarithmic plot. Outliers (< 0.9 and > 40 µg/L) were not shown for clarity.

Mastocytosis was found most frequently as a comorbidity in middle-aged patients with insect allergy (Figure 2). This comorbidity was mentioned in 4.4% of insect, 0.6% of drug, and 1.2% of food anaphylaxis cases. The basal tryptase level (outside the reaction) was evaluated in 4,243 cases (Figure 3). The value increased with age regardless of the trigger (the mean value was 5.5 µg/L in patients aged < 35 years, 6.7 µg/L in middle-aged patients, and 8.4 µg/L in those over 65 years of age (ANOVA F = 14.07, p < 0.0001). 

In particular, food-allergic patients had a previous allergic reaction (exact type of reaction was not asked for here) to the same trigger (44.6%). Patients with allergies to insects (32.8%) or drugs (22.4%) were significantly fewer. 

### Insect venom is the most common trigger of anaphylaxis in middle age 

**Figure 4:**
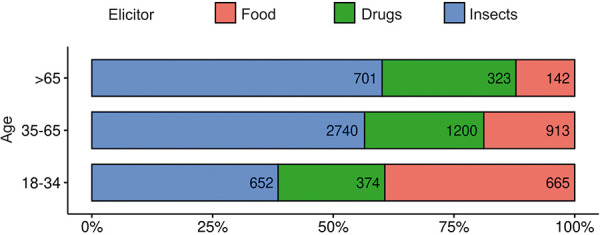
Number and percentage of reactions caused by insect stings, drugs, and food in different age groups (cases with an unknown trigger and triggers not belonging to the above categories were not included here).

In 94.8% of anaphylaxis cases in middle age, the trigger was identified, or a probable trigger was reported (data not shown). This was slightly more common than among younger patients (91%) and slightly less common than among older patients (96.5%). Food was the most common trigger in the under-35 group (34.1%) (Figure 4). Among older patients, the proportion of food-induced reactions was lower (18.8% in 35- to 65-year-olds and 11.4% in those older than 65 years). This trend was mainly related to triggers commonly associated with an atopic background (such as peanut, data not shown) rather than foods with a lower association with atopy (such as seafood, data not shown). Among middle-aged patients and those over 65 years of age, most reactions were caused by insect venom (52.1% and 56.3%, respectively) (Figure 4). Drugs were the trigger of anaphylaxis in 19.2% of those younger than 35 years, 22.8% of those 35 – 65 years, and 25.9% of those older than 65 years. 

### Reactions with skin involvement and respiratory reactions are less common in older age; cardiovascular symptoms are more common 

**Figure 5:**
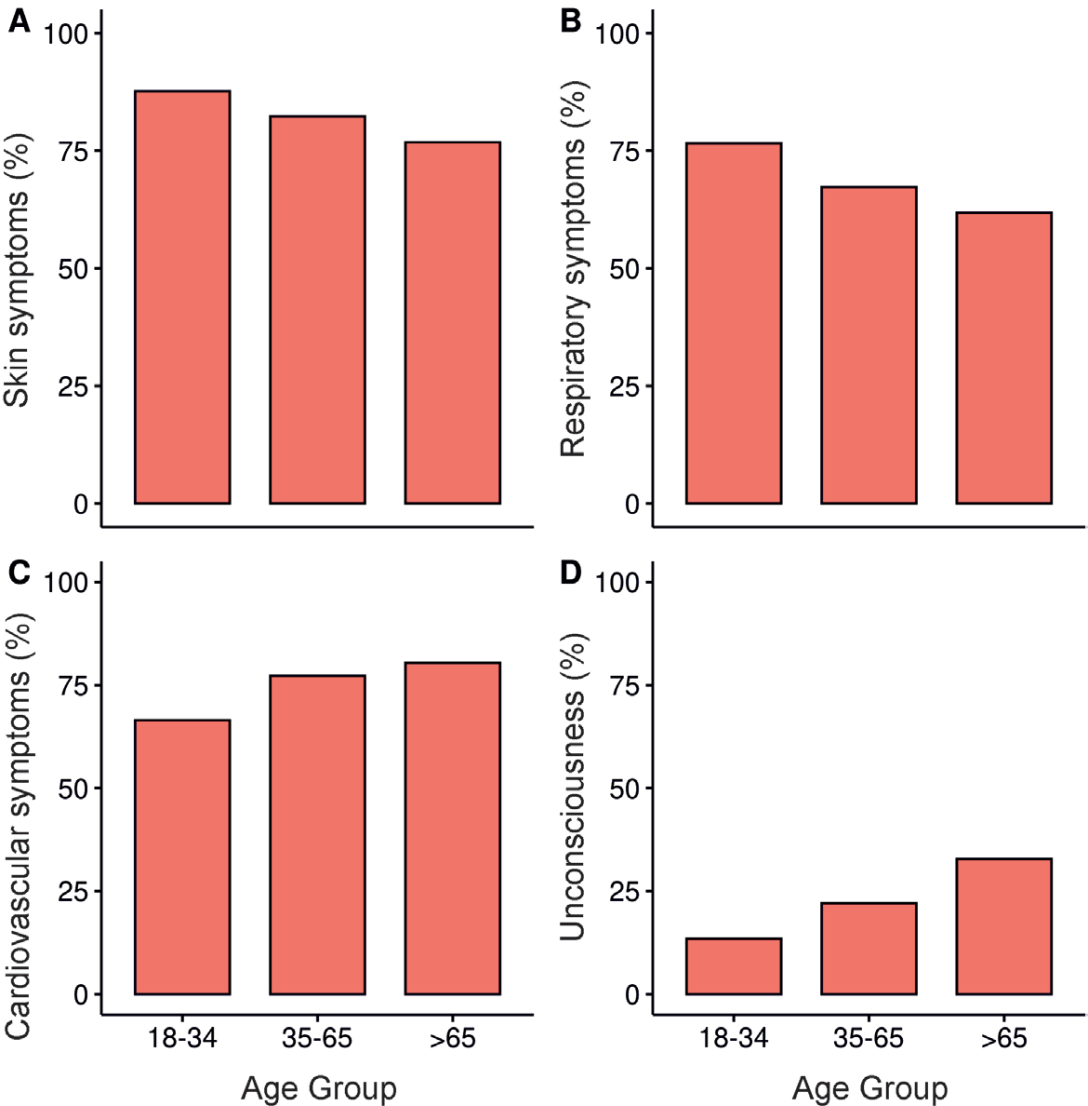
Rate of reactions with cutaneous (A), respiratory (B), and cardiovascular (C) symptoms, and loss of consciousness (D).

Skin manifestations were present in 89.3% of those younger than 35 years but only in 76.8% of those older than 65 years (Figure 5A). The proportion of reactions with respiratory symptoms also decreased with age (Figure 5B). In contrast, cardiovascular symptoms were more common in those older than 65 years (80.4%) and middle-aged persons (77.2%) than in young adults (66.5%) (Figure 5C). This tendency was particularly evident for severe cardiovascular symptoms (such as loss of consciousness) (Figure 5D). 

### Severity of reaction increases with age 

**Figure 6:**
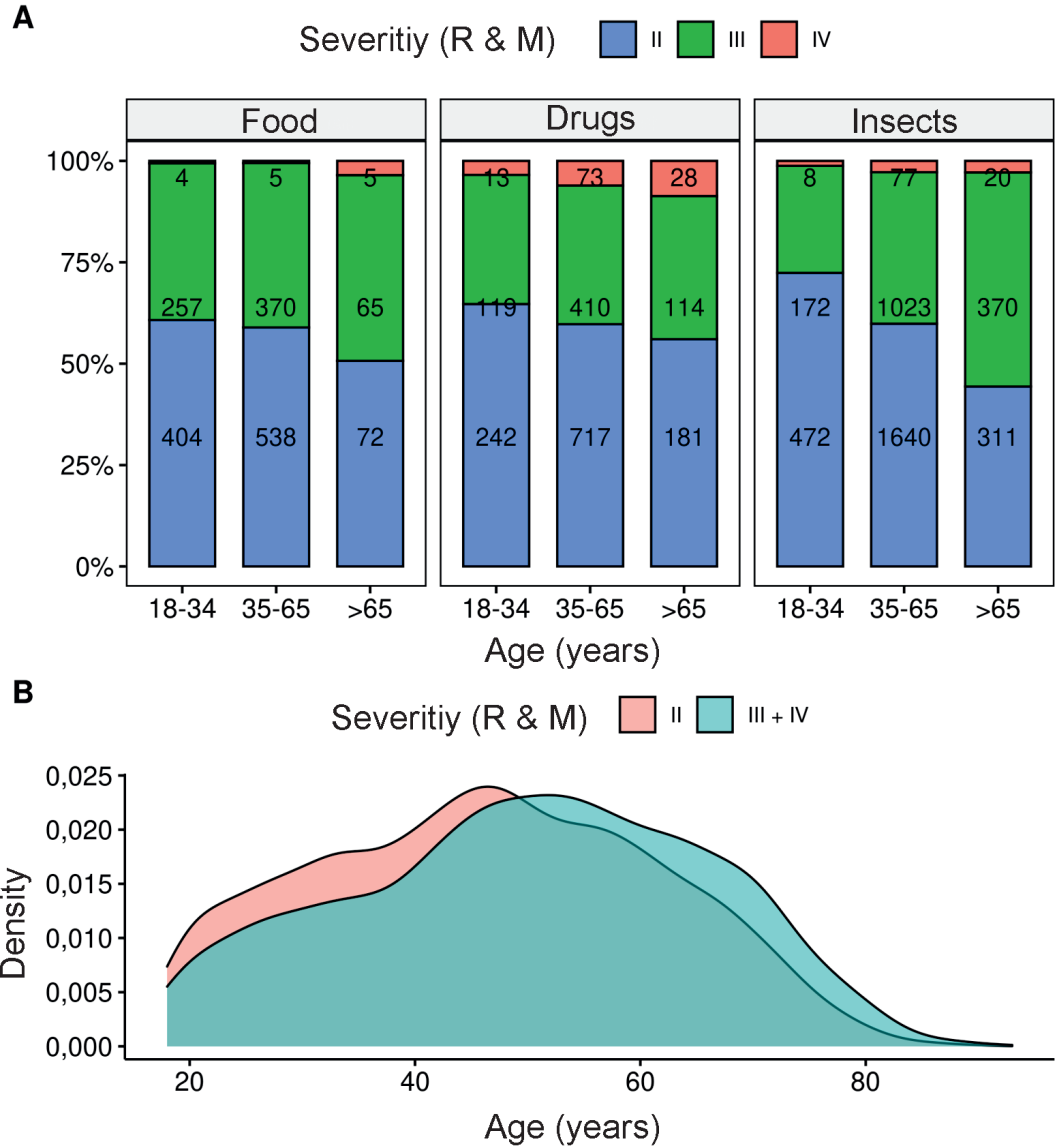
A: Number and percentage of reactions in regard to severity (according to Ring and Messmer) by age group and trigger. B: Age distribution for moderate (Ring and Messmer grade 2) or severe and very severe reactions (Ring and Messmer grade 3 + 4).

The average severity of reactions increased with age regardless of the trigger of the reaction (Figure 6). This difference was most pronounced in insect venom-allergic patients, where only 27.6% of those younger than 35 years had a severe reaction (defined as severity ≥ 3 according to Ring and Messmer), but 40.1% of middle-aged patients and 55.6% of patients older than 65 years had a severe reaction (Figure 6A). Half of all reported severe anaphylactic reactions occurred between the ages of 39 and 62 years (Figure 6B). 

## Discussion 

Our data show how triggers and symptoms of anaphylaxis change during adulthood. We have previously shown that the severity of the reaction increases with age [3, 9]. These are mostly reactions with severe cardiovascular symptoms such as loss of consciousness. An increase in cardiovascular risk factors with age on the one hand, but also the changing spectrum of triggers during the course of life might be the cause of this increasing severity. For example, hymenoptera stings as triggers are comparatively more frequently associated with severe reactions [9]. Nevertheless, these factors (age, cardiovascular risk factors, and triggers) are associated with each other, each factor also contributes independently to the severity of anaphylaxis [9]. 

The proportion of patients with atopic comorbidity decreased with age. It is known that the manifestation of atopic diseases is more frequent in the younger generation [10, 11]. It remains to be seen in the future whether today’s 20- and 30-year-olds retain their propensity for anaphylaxis to food and whether these reactions then occur more frequently with severe cardiovascular symptoms in old age (as do reactions to insect stings and medications today), or whether these reactions retain their mainly respiratory profile. 

The major limitation of our study is representativeness: because the registry recruits patients on a voluntary basis from the collaborating centers, which are unevenly distributed in Europe, the data set is not representative of the entire European population. Thus, certain types of reactions are over- or under-represented partly by design (the severe reactions) but also by the registry structure (for example, the strong over-representation of German-speaking countries and the resultant high proportion of wheat-dependent exercise-induced anaphylaxis (WDEIA), Bet v 1-dependent food allergies, and insect sting reactions and, conversely, a comparatively low proportion of peanut reactions). Therefore, comparisons with the overall population are limited. Nonetheless, we do not expect any register-related bias for age at the country levels, because within Europe access to health care is age-independent and we do not expect an age-specific restriction of the patient population at the tertiary allergy centers (pediatric cases and centers were excluded ahead). Therefore, it was surprising that in our dataset 62.2% of reactions among German adults occurred in middle-aged individuals, whereas the proportion of this age group in the adult German population is only ~ 52% [12]. Regarding young adults in Germany, the proportion observed in the registry (23%) and that expected on the basis of the total adult population (~ 22%) were almost identical. Germans over 65 years of age were significantly under-represented in our data set (14.8%) compared with the proportion of those over 65 years of age in the total adult population (~ 25%). This over-representation of middle age, although unexpected, could be due to a selection bias: in older patients, allergic reactions might be less intensively examined due to numerous other health problems or less often recognized as such due to the lack of skin symptoms. Furthermore, patients who have suffered from an allergy for decades might be better aware of the triggers and thus be able to avoid them. Another possible explanation for this numerical over-representation of middle-aged patients could result from the interaction of several factors: on the one hand, people in their middle-age years lead a very active lifestyle resulting in extensive exposure to different allergens; on the other hand, the rate of both latent and manifest previous cardiovascular damage increases in middle age. Furthermore, the generations with a higher atopic predisposition have now already reached this phase of life. The confluence of these factors may make middle age a vulnerable phase for the occurrence of anaphylaxis. Therefore, patients in this phase of life with a history of anaphylactic reaction should receive regular allergic care. Cardiovascular risk factors and pre-existing conditions should be a particular focus here, as these can be influenced to a large extent: preventive lifestyle changes and consistent optimization of drug therapy, in addition to prolonging the general life expectancy of patients [13], can reduce their risk for a severe course of anaphylaxis in middle age and beyond. 

## Conclusion 

In middle age, a change in the phenotype of anaphylaxis takes place: reactions become more severe, and cardiovascular symptoms become more frequent. Therefore, in patients with a history of anaphylaxis, triggers should be diagnosed and avoided, specific immunotherapy should be performed in the case of insect venom allergy, and preventable risks such as cardiovascular disease should be consistently reduced and treated. 

## Acknowledgment 

We thank all patients for their participation in the study and the staff of the study centers for patient recruitment and data entry: J. Grünhagen, M. Wittenberg, A. Henschel, W. Francuzik, U. Klettke, U. Staden, S. Dölle-Bierke (Berlin, Germany), A. Möser (Jena, Germany), M. Knop, E. Oppel (Munich, Germany), G. Hansen, A. Arens, B. Wedi, H. Ott (Hannover, Germany), H. Dickel (Bochum, Germany), H. Merk, S. Lehmann (Aachen, Germany), A. Köhli, B. Bogatu, K. Nemat, A. Bauer, C. Vogelberg, A. Nordwig (Dresden, Germany), V. Mahler, R. Saternus (Erlangen, Germany), E. Rietschel, N. Hunzelmann, I. Huseynow (Cologne, Germany), S. Aurich, P. Kage, F. Prenzel, J. Zarnowski (Leipzig, Germany), L. Klimek, O. Pfaar (Wiesbaden, Germany), N. Reider (Innsbruck, Austria), W. Aberer (Graz, Austria), F. Riffelmann, M. Wenzel (Schmallenberg, Germany), T. Kinaciyan, Z. Szepfalusi, C. Ebner, F. Horak (Vienna, Austria), R. Brehler (Münster, Germany), J. Witte, S. Hompes, C. Kemen, P. Stock, (Hamburg, Germany), L. Lange, T. Bieber, M. Bücheler (Bonn, Germany), U. Rabe (Treuenbritzen, Germany), P. Schmid-Grendelmeier, C. Stadlin, M. Hoernes, (Zurich, Switzerland), W. Brosi (Würzburg, Germany), S. Nestoris (Lippe-Lemgo, Germany), R. Bruns (Greifswald, Germany), E. Varga (Graz, Austria), P. Eng (Aarau/Lucerne, Switzerland), T. Reese (Rheine, Germany), M. Polz (Ruesselsheim, Germany), S. Schweitzer-Krantz, S. Meller (Düsseldorf, Germany), H. Rebmann, J. Fischer (Tübingen, Germany), T. Spindler (Davos, Switzerland), G. Stichtenoth (Lübeck, Germany), S. Thies (Schwedt, Germany), I. Yildiz (Neumünster, Germany), M. Gerstlauer (Augsburg, Germany), P. Utz (Wangen im Allgäu, Germany), J. Klinge (Fürth, Germany), S. Volkmuth (Velbert, Germany), S. Plank-Habibi (Alzenau, Germany), B. Schilling (Passau, Germany), A. Brückner, A. Kleinheinz (Buxtehude, Germany), K. Schäkel, S. Hämmerling (Heidelberg, Germany), NG. Papadopoulos, I. Manolaraki (Athens, Greece), M. Kowalski (Lodz, Poland), K. Solarewicz-Madajek (Wroclaw, Poland), C. Körner-Rettberg (Bochum, Germany), S. Tscheiller, P. Beaumont, D. Sabouraud, G. Pouessel, É. Beaudouin, JM. Renaudin and members of the Réseau d’Allergo-Vigilance (France), T. Mustakov, G. Christoff (Sofia, Bulgaria), J. Seidenberg (Oldenburg, Germany), N. Cabañes Higuero (Toledo, Spain), A. Vega Castro (Guadalajara, Spain), S. Büsing (Osnabrück, Germany), C. Virchow (Rostock, Germany), A. Gülsen, U. Jappe (Borstel, Germany), H. Straube (Darmstadt, Germany), S. Müller (Freibung, Germany), F. Knöpfel (Norderney, Germany), B. Rogala (Zabrze, Poland), A. Montoro, M. Fernandez-Rivas, TM. De Vicente Jiménez (Madrid, Spain), A. Brandes (Frankfurt/Oder, Germany), A. Muraro (Padua, Italy), T. Buck, J. Büsselberg (Hannover-Misburg, Germany), N. Zimmermann (Potsdam, Germany), D. Hernandez (Valencia, Spain), P. Minale (Genoa, Italy), J. Niederwimmer and B. Zahel (Linz, Austria), A. Fiocchi (Rome, Italy), A. Reissig (Gera, Germany), (Düsseldorf, Germany), F. Eitelberger (Wels, Austria), R. Asero (Milan, Italy), F. Hermann, S. Zeidler (St. Augustin, Germany), S. Pistauer (Sylt/Westerland, Germany), M. Geißler (Ribnitz-Damgarten, Germany), E. Cichocka-Jarosz, I. Tarczon (Krakow, Poland), LF Ensina (Sao Paulo, Brazil), JOB Hourihane, I. Maris (Cork, Ireland), I. Iwona Poziomkowska-Gęsicka (Szczecin, Poland), A. Plaza Martin, V. Cardona (Barcelona, Spain), J. Meister (Aue, Germany), B. Kreft (Halle (Saale), Germany), MB Bilò (Ancona, Italy), BE. García (Pamplona, Spain), S. Stieglitz (Wuppertal, Germany), I. Neustädter (Nuremberg, Germany), and E. Hamelmann (Bielefeld, Germany). 

## Funding 

The anaphylaxis register is organized and supported as part of the Network for Online Registration of Anaphylaxis (NORA e.V.). 

## Conflict of interest 

MW declares honoraria for lectures and consulting from ALK-Abelló Arzneimittel GmbH, Mylan Germany GmbH, Leo Pharma GmbH, Sanofi-Aventis Deutschland GmbH, Regeneron Pharmaceuticals, DBV Technologies S.A, Stallergenes GmbH, HAL Allergie GmbH, Allergopharma GmbH & Co.KG, Bencard Allergie GmbH, Aimmune Therapeutics UK Limited, Actelion Pharmaceuticals Deutschland GmbH, Novartis AG, Biotest AG, AbbVie Deutschland GmbH & Co. KG and Lilly Deutschland GmbH. RT received honoraria for lectures and consulting services from ALK-Abello. CP performed clinical trials for Novartis, BMS, and Allergy Therapeutics and received honoraria for lectures and consultations from Novartis, BMS, Allergy Therapeutics, Roche, Pierre Fabre, SUNPHARMA, MSD, and Sanofi Genzyme. RL received travel support from Bencard, ALK-Abelló, and Thermo Fisher Scientific. The other authors declare no conflict of interest with respect to this publication.
